# Investigation of the effects of radiotherapy and chemotherapy on brain volume in cancer patients: brain tumor study

**DOI:** 10.1007/s11060-025-05366-6

**Published:** 2026-02-13

**Authors:** Birgül Deniz, Muhammed Furkan Arpaci, Hıdır Pekmez, Gökçe Bağci Uzun, Feyza İnceoğlu, Hakan Harputluoğlu

**Affiliations:** 1 Department of Medical Oncology, Faculty of Medicine, Inonu University, Malatya Elazig Road 10. km No:44210, Battalgazi, Malatya, 44000 Turkey; 2 Department of Anatomy, Faculty of Medicine, Malatya Turgut Özal University, Malatya, Turkey; 3 Department of Biostatistics, Faculty of Medicine, Malatya Turgut Özal University, Malatya, Turkey; 4 Department of Oncology, Faculty of Medicine, Inonu University, Malatya, Turkey

**Keywords:** Brain tumor volume, Radiotherapy, Chemotherapy, Brain volume, Telencephalon, Diencephalon

## Abstract

**Purpose:**

Brain tumors, characterized by the uncontrolled proliferation of abnormal cells within cerebral tissue, remain clinically challenging entities. Radiotherapy and chemotherapy constitute fundamental therapeutic modalities; however, their effects on healthy brain structures are not fully understood. This study aimed to evaluate the impact of these treatments on volumetric changes in brain structures and tumor size in patients with primary or metastatic brain tumors.

**Methods:**

A retrospective cohort of 47 patients aged 18–90 years treated at Inonu University Turgut Özal Medical Center between 2012 and 2023 was analyzed. Brain MRI scans were evaluated at three time points: pre-treatment, post-radiotherapy, and post-chemotherapy. Radiotherapy was delivered at a median dose of 60 Gy in 30–33 fractions, and temozolomide was used as the chemotherapy agent. Volumetric measurements of the telencephalon, diencephalon, ventricles, white matter, brainstem, cerebellum, and cerebral cortex were performed using MRICloud, while tumor volumes were quantified using the VolBrain platform. All volumetric differences were statistically tested using repeated-measures ANOVA with corresponding p-values reported.

**Results:**

A statistically significant increase in telencephalon volume was observed after radiotherapy, followed by a return toward baseline measurements after chemotherapy. The diencephalon demonstrated a significant and persistent volume reduction following radiotherapy (*p* < 0.05). No statistically significant volumetric changes were identified in the ventricles, white matter, brainstem, cerebellum, or cerebral cortex (*p* > 0.05). Tumor volume changes were also statistically evaluated and showed no significant differences across the three time points (*p* = 0.456), indicating stable disease during the treatment course.

**Conclusion:**

Radiotherapy and chemotherapy lead to region-specific volumetric alterations in the brain. The transient telencephalon enlargement is more likely attributable to treatment-related edema or inflammatory processes rather than functional improvement. The persistent diencephalon volume decline may reflect early treatment-related tissue vulnerability. Incorporating automated volumetric assessment into routine follow-up may support early detection of therapy-related structural changes and facilitate more personalized treatment planning.

## Introduction

Brain tumors are lesions formed by the uncontrolled proliferation of abnormal cells within brain tissue. These tumors are classified as primary tumors that originate directly from brain tissue or secondary (metastatic) tumors that metastasize to the brain parenchyma from another part of the body [1]. Brain tumors are divided into several types, including gliomas, meningiomas and metastatic tumors. Gliomas are the most common type of brain tumor and include several histological forms [2]. Brain tumors, which are classified as benign (benign) or malignant (malignant), may cause various clinical symptoms depending on factors such as tumor size, location and growth rate [3].

Brain tumors may affect different structures depending on their location. For instance, they may block the flow of cerebrospinal fluid between the ventricles and cause cerebral edema as a result of the accumulation of this fluid. Cerebral edema may lead to various clinical manifestations such as headache, epileptic seizures and focal neurological deficits [4]. Moreover, depending on the lobes they affect, brain tumors may cause various neurological and cognitive symptoms such as behavioral changes, motor disorders, pain, memory loss, loss of motor coordination, speech difficulties, headaches, seizures, visual and hearing impairments, and impaired memory functions [2, 5–7].

Brain tumors are among the major health problems that can cause serious morbidity and mortality in all age groups. Although various studies have been conducted for many years to elucidate the etiology of these tumors, a clear and distinct risk factor that could explain the majority of cases has not yet been identified [8]. The only environmental risk factor reported in the literature regarding the etiology of brain tumors is exposure to ionizing radiation. Meningiomas and gliomas that develop due to ionizing radiation exposure are more common in younger age groups [9]. Brain tumors are reported to be the eighth most common type of cancer in adults over the age of 40. A significant proportion of brain tumors diagnosed in individuals aged 20 years and older are malignant. However, the age-adjusted incidence of malignant brain tumors in this age group is relatively low at approximately 8.5 per 100,000 individuals [10].

Primary brain tumors are one of the leading causes of cancer-related deaths in high-income countries [11]. Primary brain tumors are classified histologically according to the tissues from which they originate, and this classification is based on the criteria set by the World Health Organization (WHO). In the light of current research, staging of primary brain tumors plays an important role in determining the treatment options to be applied. Clinical data such as patient age, environmental factors, tumor localization and radiological findings and pathological parameters such as extent of surgical resection, proliferation indices and genetic mutations are critical in determining the prognosis [4].

Brain tumors constitute a complex group of diseases that need to be managed with a multidisciplinary approach. The most common primary brain tumor is glioblastoma multiforme, which accounts for approximately half of all primary brain tumors. Although surgical resection is attempted in all patients, it is usually not sufficient alone. Therefore, adjuvant radiotherapy and chemotherapy have an important place in the treatment process [12]. Surgical treatment aims to remove the tumor completely when possible, while radiotherapy involves the use of high-energy beams to destroy tumor cells. Chemotherapy involves the administration of various drugs to slow the growth of the tumor and destroy cancer cells. Especially in aggressive and fast-growing tumors such as glioblastoma, chemotherapy and radiotherapy are often combined to achieve a faster and more effective treatment response [13]. It is recommended that patients be followed up with magnetic resonance imaging (MRI) every 3 to 6 months for the first five years and annually thereafter [14].

Determining the histological features of brain tumors plays a critical role in both planning the treatment process and predicting the prognosis of the disease. Conventional magnetic resonance imaging (MRI) sequences provide important information for the diagnosis and evaluation of anatomical details of tumors [15]. In addition, volumetric measurements of brain structures such as the cerebellum, cerebrum, nucleus caudatus, globus pallidus, thalamus, corpus amygdaloideum and hippocampus and tumor volumes can be evaluated in cubic centimeters (cm³) through open access online data-based applications such as VolBrain and MRICloud [16, 17].

This study aims to objectively measure brain structures and tumor volumes in patients diagnosed with brain tumors before and after treatment using VolBrain and MRICloud systems. The findings aim to contribute to a deeper understanding of the relationship between changes in brain structures and the course of the disease and the treatment process. It also aims to reveal the potential uses of these systems in clinical applications. Such objective and quantitative measurements may enable the creation of individualized treatment plans for patients and thus contribute to improving quality of life.

## Methods

The research was conducted in the Medical Oncology Departments of Inonu University Turgut Özal Medical Center. In our study, the records of the patients who came to the Medikal Onkoloji Department between 2012 and 2023 were examined. Ethical approval was obtained with the decision of Malatya Turgut Özal University Non-Invasive Clinical Research Ethics Committee dated 14.06.2023 and numbered 2022/193.

This study was performed retrospectively on brain magnetic resonance (MR) images of patients aged 18–90 years who were diagnosed with intracranial masses and treated with radiotherapy and/or chemotherapy.

Our study included brain MRI images and epicrisis files of 500 patients diagnosed with intracranial masses between the specified dates. However, 400 patients without brain MRI images before or after radiotherapy or chemotherapy, 30 patients without FLAIR sequence and 23 patients with severe motion artifact were excluded.

**Fig. 1:**
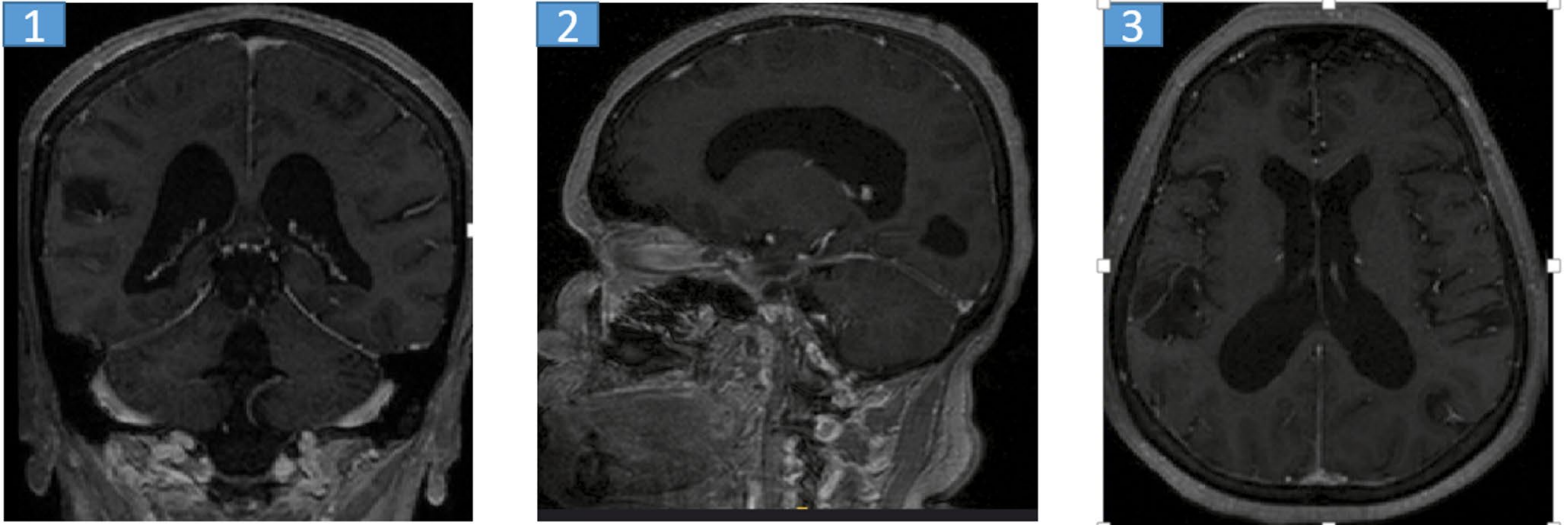
MRI scan of the brain in a 50-year-old female patient diagnosed with a tumor **1**: Coronal MRI image of the brain with tumor. **2**: Sagittal MRI image of the brain with tumor **3**:Axial MRI image of the brain with tumor

**Fig. 2:**
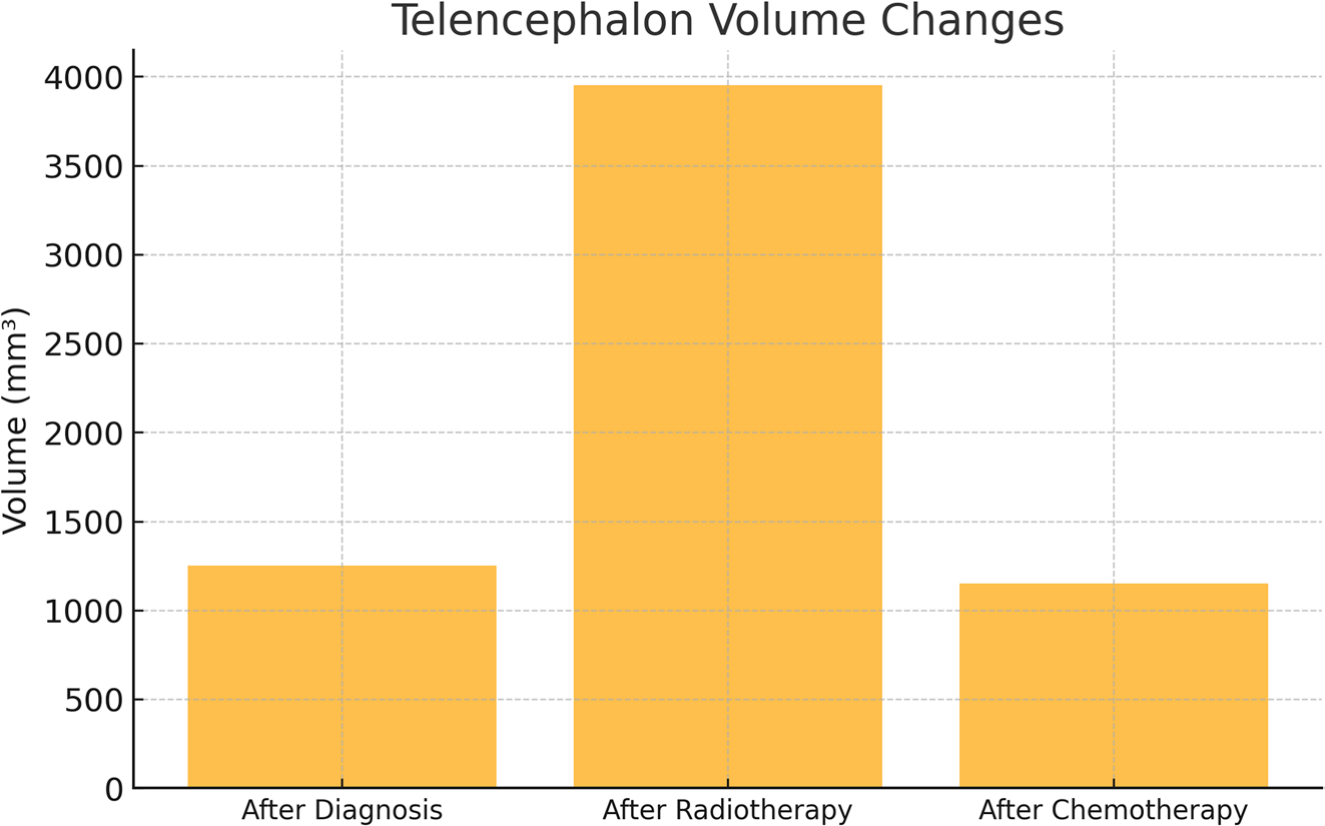
Telencephalon volume changes across treatment stages

**Fig. 3:**
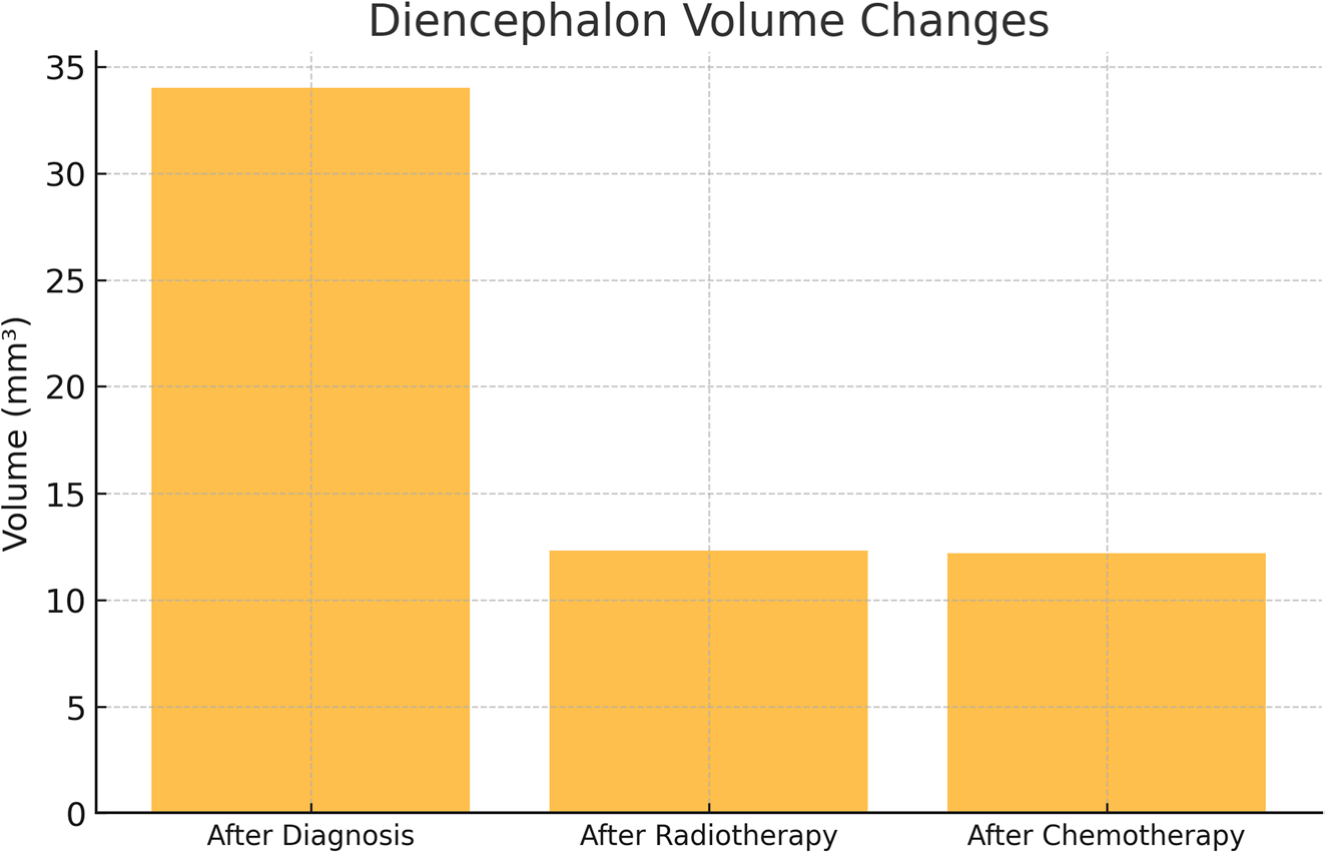
Diencephalon volume changes across treatment stages

A total of 47 patients (11 females, 36 males) with primary or metastatic brain tumors who met the inclusion criteria were included in the study. The radiotherapy techniques applied to these patients were analyzed by a medical physicist (JP) and necessary calculations were performed using simulator images of the treatment field setup and treatment records during the evaluation process.The three-dimensional dose distribution for each patient was calculated using a standard computed tomography (CT) set (CadPlan 3.1.1, Varian Oncology Systems, 1998, Espoo, Finlandiya). The median total radiotherapy dose was 60 Gy in 30 to 33 fractions, with a dose range of 56 to 68 Gy. Nineteen patients received whole brain irradiation at a dose of 40 Gy from two contrasting fields, followed by a boost dose ranging from 20 to 28 Gy to the tumor bed. Nine patients received only focused (focal) irradiation, and the median total dose in this group was 60 Gy (range: 56–66 Gy) in 28 to 34 fractions. Patients who received whole brain irradiation received adjuvant chemotherapy with temozolomide after radiotherapy (Figs. 1, 2 and 3).

Patients with incomplete treatment, missing at least one of the pre-treatment, post-chemotherapy or post-radiotherapy magnetic resonance imaging (MRI) images, history of head trauma, intracranial surgery and patients with a different neurological disease before diagnosis were excluded from the study. The brain MR images of 47 patients with intracranial masses before treatment (first measurement), after radiotherapy (second measurement) and after chemotherapy (third measurement) were evaluated and analyzed by grouping the patients according to these three measurement times.

Magnetic resonance (MR) imaging of all patients was performed at Inönü University Turgut Özal Medical Center Department of Radiology using a 1.5 Tesla Siemens field scanner (Siemens Healthineers GmbH, Erlangen, Germany) equipped with a 32-channel head coil. MPRAGE (Magnetization Prepared Rapid Acquisition Gradient Echo) sequence prepared with T1-weighted three-dimensional magnetization was used for volumetric analysis. Images; TR: 2300 ms, TE: 2.32 ms, field of view (FOV): 240 mm, matrix size: 256 × 256 and slice thickness: 0.9 mm.

Following the scanning procedure, the volumetric image data were downloaded and transferred to a personal computer. In order to calculate brain volumes, certain analysis steps were followed respectively. First, the image data were converted into title (HDR) and image (IMG) formats using DTIStudio software. The resulting HDR and IMG files were uploaded to MRICloud, a free web-based module that works remotely and performs automated volumetric analysis, providing volumetric information for each submitted case. MRICloud is a fully automated cloud service for segmentation of brain images of MPRAGE sequences based on multiple atlas probabilities. The fusion algorithm used in this process was based on the atlas adult_286labels_11atlases_V5L, a multi-atlas inventory of 286 anatomical structures developed by Johns Hopkins University [18, 19]. The volumetric analysis results were automatically calculated by the system and the data for each measurement time were presented in separate files. The data obtained were classified as “post-diagnosis”, “post-radiotherapy” and “post-chemotherapy” for each patient and saved in an Excel file.

The volbrain program was used to measure the tumor volumes of the patients. VolBrain is a free system that works automatically over the internet (http://volbrain.upv.es/), allowing brain volumes to be measured without human interaction. It automatically performs volumetric brain analysis on T1-weighted images. T1-layer images of the patient were selected from the computer and extracted in DICOM format and saved in a separate folder on the desktop. The dcm2niigui file was opened from the Mricron file. The folder with images with Dcm extension was dragged to the dcm2nii application. Compressed FSL (4D NıfTI nii) was selected as output format. DCM2NII converted the DICOM images to the NifTI format used by the brain imaging tool. The NlfTI file was converted to winrar file and added to the archive. Vol 2 Brain was typed into the search engine [20]. The NifTI file added to the archive was selected from the select file section, and the results of the person whose age and gender information was entered were saved as pdf to the user’s e-mail within a few minutes. The patient’s results were recorded in the excel file as post-diagnosis, post-radiotherapy and post-chemotherapy.

In our study, MRICloud was used to analyze the volumes of the telencephalon, diencephalon, ventricles, white matter, brainstem, cerebellum, and cerebral cortex. In addition, tumor volumes were evaluated using the VolBrain platform.

### Statistical analysis

The sample size of the study was determined by power analysis. In the calculation made using the G*Power 3.1 program, the required sample size was calculated as 39 based on an effect size of 0.55, 5% margin of error (α = 0.05), 95% confidence level and 95% population representativeness (power = 0.95). Cohen defines an effect size of 0.40 and above as a high effect size. The effect size was determined based on Cohen’s coefficients [21].

Statistical analyses of the data included in the study were performed using SPSS (Statistical Package for the Social Sciences) version 25 program. The Kolmogorov-Smirnov test was used to evaluate whether the data fit the normal distribution. Significance level was accepted as *p* < 0.05 in comparison tests. In cases where the variables were found to be normally distributed (*p* > 0.05), the analyses were carried out with parametric test methods.

In the comparison of dependent dyadic groups, paired samples t-test was applied since the normality assumption was met. Repeated measures analysis of variance (Repeated Measures ANOVA) was used to evaluate the difference between groups based on repeated measures. Multiple normal distribution and homogeneity of variance assumptions were checked before the analysis. Mardia’s Multivariate Normality Test was used for multivariate distribution. Mauchly’s Test of Sphericity was used for the sphericity test, Levene’s test was used for variance homogeneity between groups, and Box’s M Test was used for the equality of covariance matrices.

## Results

A total of 47 patients participated in the study, with a median age of 53.89 years (range: 19–88). When the volumetric changes of brain structures were evaluated across the three measurement sessions, significant differences were observed in the telencephalon and diencephalon, whereas the ventricles, white matter, brainstem, cerebellum, cerebral cortex, and tumor volume showed no statistically significant changes.

A statistically significant difference was found among the telencephalon measurements (F = 41.909, *p* = 0.001) (Table 1).

**Table 1:** Comparison of measurements

Variable	After Diagnosis (First Measurement)	After radiotherapy (Second Measurement)	After Chemotherapy (third Measurement)	Intergroup Comparison (F)		Within Group Comparison (t)	
	Mean ± sd	Mean ± sd	Mean ± sd				
Telencephalon	1245,47 ± 595,76	3941,01 ± 2824,89	1157,24 ± 516,28	***F = 41***,***909***	*p* = 0,001*	First-second	*p* = 0,001*
						First-third	*p* = 1,000
						Second-third	*p* = 0,001*
	Effect size (η^2^) = 0.491						
Diencephalon	33,85 ± 26,22	12,28 ± 6,63	12,25 ± 6,22	***F = 28***,***387***	*p* = 0,001*	First-second	*p* = 0,001*
						First-third	*p* = 0,001*
						Second-third	*p* = 1,000
	Effect size (η^2^) = 0.398						
Ventricle	47,96 ± 28,79	38,72 ± 28,56	44,38 ± 33,03	F = 1,403	*p* = 0,251	First-second	*p* = 0,238
						First-third	*p* = 1,000
						Second-third	*p* = 0,987
	Effect size (η^2^) = 0.066						
White Matter	626,86 ± 462,44	545,4 ± 341,38	562,53 ± 317,72	F = 0,683	*p* = 0,505	First-second	*p* = 0,842
						First-third	*p* = 1,000
						Second-third	*p* = 1,000
	Effect size (η^2^) = 0.026						
Brainstem	37,19 ± 23,14	28,76 ± 18,63	31,13 ± 18,31	F = 2,75	*p* = 0,071	First-second	*p* = 0,122
						First-third	*p* = 0,331
						Second-third	*p* = 1,000
	Effect size (η^2^) = 0.091						
Cerebellum	208,13 ± 165,46	152,4 ± 96,87	173,17 ± 91,34	F = 2,597	*p* = 0,088	First-second	*p* = 0,134
						First-third	*p* = 0,612
						Second-third	*p* = 0,855
	Effect size (η^2^) = 0.088						
Cerebral Cortex	599,92 ± 222,7	543,59 ± 240,44	578,33 ± 218,36	F = 1,004	*p* = 0,368	First-second	*p* = 0,603
						First-third	*p* = 1,000
						Second-third	*p* = 1,000
	Effect size (η^2^) = 0.036						
Total tumor volume	12,8 ± 14,62	10,79 ± 8,04	16,54 ± 40,87	F = 0,622	*p* = 0,456	First-second	*p* = 0,962
						First-third	*p* = 1,000
						Second-third	*p* = 1,000
	Effect size (η^2^) = 0.038						

*SD: standard deviation; F: repeated measures ANOVA test value; t: dependent t-test; *p* < 0.05 indicates a significant statistical difference between the measurements

The telencephalon volume increased markedly after radiotherapy, showing nearly a threefold rise compared to baseline, and subsequently decreased during chemotherapy, returning to a level comparable to the initial measurement. This pattern suggests that radiotherapy may have induced transient edema or inflammatory enlargement, which resolved in the later treatment phase.

A statistically significant difference was also detected in diencephalon measurements (F = 28.387, *p* = 0.001) (Table 1).

The diencephalon volume significantly decreased after radiotherapy, and this reduction remained stable during chemotherapy. Since there was no difference between the second and third measurements, the structural loss appears primarily related to radiotherapy-induced atrophy rather than ongoing treatment effects. This finding is consistent with prior studies reporting early volume reductions in deep gray matter structures following radiotherapy.

The ventricles (*p* = 0.251), white matter (*p* = 0.505), brainstem (*p* = 0.071), cerebellum (*p* = 0.088), and cerebral cortex (*p* = 0.368) did not show statistically significant differences across the three time points. Although minor fluctuations were observed, these variations did not reach statistical significance.

White matter and cortical changes often emerge in the late-delayed period after radiotherapy; therefore, longer follow-up may reveal more pronounced alterations.

There was no statistically significant difference in tumor volume across the first, second, and third measurements (F = 0.622, *p* = 0.456).

Although tumor volume showed increases and decreases between the measurements, these fluctuations were not statistically significant. This finding suggests that the effect of treatment may not manifest in tumor volume during the early period, or that a measurable radiological response may require longer follow-up. Therefore, the tumor volume was statistically tested, and it was determined that treatment did not produce a significant change in tumor size (*p* = 0.456).

The effect size for the telencephalon was 0.491, indicating that the time variable explained 49.1% of the variance in telencephalon volume. The effect size for the diencephalon was 0.398, showing that the time variable accounted for 39.8% of the variance in diencephalon volume.21.

## Discussion

In this study, the findings were interpreted in the context of previous research, with a particular focus on evaluating the early effects of radiotherapy and chemotherapy on brain structures, and discussed in comparison with the existing literature to highlight their potential short- and long-term impacts.

In addition, the accuracy and reproducibility of the automated volumetric tools used in this study should be considered. MRICloud has been validated in various neurological conditions, including stroke, multiple sclerosis, and neurodegenerative diseases, showing reliable segmentation even in the presence of anatomical distortion, edema, and mass effect [22]. Similarly, VolBrain has demonstrated high agreement compared with manual segmentation and other automated platforms, with studies confirming its robustness in both healthy and pathological brains [23, 24]. However, segmentation errors may still occur in tumor-adjacent structures or in regions affected by treatment-related changes, and this limitation should be taken into account when interpreting volumetric findings in tumor-affected anatomy.

Additionally, tumor volume was also assessed and compared with previous findings, allowing for a more comprehensive evaluation of treatment response alongside structural brain changes.

Radiotherapy (RT) and chemotherapy are vital therapeutic approaches in the treatment of head and neck tumors and brain tumors [25]. Although radiotherapy planning and delivery techniques have improved significantly in recent years, radiation exposure of healthy tissues around the target lesion cannot be completely avoided [26]. This situation necessitates careful evaluation of treatment efficacy as well as treatment-related complications. These findings suggest that despite the technical advances made in the field of radiotherapy, it still carries significant risks for sensitive brain regions.

The negative prognosis of brain tumors despite modern treatment protocols suggests that it is not sufficient to limit treatment strategies to tumor targeting alone. Moreover, several studies have shown that chemotherapy added to radiotherapy does not significantly improve survival [27]. This situation requires that treatment strategies should be evaluated not only in terms of survival time, but also in terms of quality of life and preservation of cognitive functions. These results clearly demonstrate the need for new and holistic approaches that can be integrated into existing treatments.

It was reported in various studies that brain volume reduction occurs after radiotherapy and chemotherapy in patients diagnosed with Glioblastoma Multiforme (GBM) [28, 29]. In our study, especially the telencephalon volume was examined in detail; a significant increase was observed in the measurements made after radiotherapy, but no statistically significant difference was found between the measurements before and after chemotherapy (*p* < 0.05). This finding suggests that edema or inflammatory responses due to radiotherapy may cause a transient increase in volume.

The edema that forms in the brain tissue due to radiotherapy can lead to temporary increases in volume. During chemotherapy, it is possible for the brain volume to return to normal levels with the regression of this edema. The increase in telencephalon volume observed after RT can be explained by this physiopathologic mechanism. This observation suggests that early volume increases should not be mistakenly interpreted as tumor progression.

Statistically significant volume losses were also observed in the measurements made in the diencephalon region (*p* < 0.05). While there was a significant difference between the measurements made before and after both radiotherapy and chemotherapy, there was no significant difference between the measurements made after radiotherapy and after chemotherapy. This finding suggests that radiotherapy may cause permanent effects on the diencephalon region and is consistent with the studies of Nagtegaal et al. [30]. This suggests that the diencephalon region may be of special importance in terms of its sensitivity to radiotherapy.

In the literature, it is reported that ventricular enlargement after radiotherapy usually occurs in the late period [29, 31]. However, in this study, no statistically significant change in ventricular volume was detected because imaging procedures were performed early after treatment. This indicates that the results obtained were evaluated independently of late effects and in a manner free from external factors. This finding supports that early imaging may not reflect late complications but may more reliably show treatment-specific effects.

In studies conducted on white matter, some research emphasizes volume loss [32], while others have not detected any significant changes [29]. In this study as well, no significant change in white matter volume was observed (*p* >0.05). The findings suggest that white matter volume can be preserved in the short and medium term. This result indicates that white matter may be relatively resistant to treatment, but highlights the necessity of long-term follow-up.

Changes in white matter volume may be affected by individual factors such as age, genetic predisposition and comorbidities. Therefore, long-term studies with larger samples are needed for a more reliable and generalized assessment of these changes. This interpretation reveals that the effect of individual differences on brain structure is too important to be ignored.

In terms of ventricular volume, although lateral ventricular enlargement has been reported in the literatüre [33] no significant change was found in our study. This study is important as it is one of the rare examples performed in the early period. The findings suggest that changes in ventricular volume are time sensitive and that these effects may not be observed in the early period.

In one of the limited number of studies on the cerebellum, Raschke et al. reported a decrease in volume in this region. However, no statistically significant change in cerebellum volume was found in the present study [34]. The findings regarding the cerebellum are contradictory in the literature and more and more comprehensive studies are needed to clarify the response of this anatomical region to treatment. The results obtained show that the response of the cerebellum to treatment is still unclear and further research is needed to resolve the uncertainties in this area.

Seibert et al. reported a dose-dependent decrease in cerebral cortex volume after chemoradiotherapy [35]. But in this study, no statistically significant change in cerebral cortex volume was detected since a fixed dose protocol was applied. This finding emphasizes that volume changes may be sensitive to the administered treatment dose and dose-variation analyses should be considered in clinical evaluations.

Previous studies on the brain stem have reported no volume change in this region after radiotherapy [36]. Similarly, in the present study, no statistically significant change was found in brainstem volume after radiotherapy and chemotherapy. This finding is consistent with the existing data in the literature and suggests that the brainstem may be relatively resistant to chemoradiotherapy. This suggests that the brainstem may maintain its structural integrity in the short term and exhibit a more stable anatomical structure against treatment.

Lastly, studies conducted by Ellingson and Oshima showed a significant reduction in tumor volume after treatment [37, 38]. But in the present study, no significant reduction in tumor volume was observed; only tumor progression was halted. This finding suggests that treatment efficacy should be assessed not only by tumor volume reduction but also by prevention of tumor progression.

The first limitation of the study is the mortality of the patients in the period of measuremets, the second limitation of the study is the disruption of the MRI when evaluating the measurements.

## Data Availability

The dataset used and/or analyzed during the current study available from the corresponding author on reasonable request.
